# Intra-Abdominal Fat Depots Represent Distinct Immunomodulatory Microenvironments: A Murine Model

**DOI:** 10.1371/journal.pone.0066477

**Published:** 2013-06-12

**Authors:** Courtney A. Cohen, Amanda A. Shea, C. Lynn Heffron, Eva M. Schmelz, Paul C. Roberts

**Affiliations:** 1 Department of Biomedical Sciences and Pathobiology, Virginia Polytechnic Institute and State University, Blacksburg, Virginia, United States of America; 2 Department of Human Nutrition, Foods and Exercise, Virginia Polytechnic Institute and State University, Blacksburg, Virginia, United States of America; University of Cincinnati, United States of America

## Abstract

White adipose tissue (WAT) is a multi-faceted endocrine organ involved in energy storage, metabolism, immune function and disease pathogenesis. In contrast to subcutaneous fat, visceral fat (V-WAT) has been associated with numerous diseases and metabolic disorders, indicating specific functions related to anatomical location. Although visceral depots are often used interchangeably in V-WAT-associated disease studies, there has been a recent subdivision of V-WAT into “true visceral” and non-visceral intra-abdominal compartments. These were associated with distinct physiological roles, illustrating a need for depot-specific information. Here, we use FACS analysis to comparatively characterize the leukocyte and progenitor populations in the stromal vascular fraction (SVF) of peritoneal serous fluid (PSF), parametrial (pmWAT), retroperitoneal (rpWAT), and omental (omWAT) adipose tissue from seven-month old C57BL/6 female mice. We found significant differences in SVF composition between all four microenvironments. PSF SVF was comprised almost entirely of CD45^+^ leukocytes (>99%), while omWAT contained less, but still almost two-fold more leukocytes than pmWAT and rpWAT (75%, 38% and 38% respectively; p<0.01). PmWAT was composed primarily of macrophages, whereas rpWAT more closely resembled omWAT, denoted by high levels of B1 B-cell and monocyte populations. Further, omWAT harbored significantly higher proportions of T-cells than the other tissues, consistent with its role as a secondary lymphoid organ. These SVF changes were also reflected in the gene expression profiles of the respective tissues. Thus, intra-abdominal fat pads represent independent immunomodulatory microenvironments and should be evaluated as distinct entities with unique contributions to physiological and pathological processes.

## Introduction

White adipose tissue (WAT) is the largest endocrine organ in the body, comprising up to 45% of total body composition in obese individuals (BMI≥30). Once thought to be a passive reservoir for excess energy storage, WAT is increasingly recognized for its role in metabolism, immune and endocrine function, thermoregulation, and tissue repair [1]. Its function in both physiological and pathological processes may be influenced by either tissue resident adipocytes or the stromal vascular fraction (SVF), which includes leukocytes, mesenchymal stem cells, adipocyte progenitors, fibroblasts, and endothelial cells [2]. Recent studies indicate functional differences in adipose tissue depots related to anatomical location, specifically between subcutaneous fat (S-WAT) and visceral fat (V-WAT). While expansion of S-WAT is associated with improved insulin sensitivity and decreased risk of type 2 diabetes, V-WAT expansion is linked to an increased risk of cardiovascular disease, glucose dysregulation, hypertension, and certain cancers [3], [4]. Functional and secretory differences in V-WAT as compared to S-WAT include increased lipolysis and expression of inflammatory molecules, and decreased angiogenesis, production of adiponectin and leptin, and responsiveness to insulin [5]. Although V-WAT comprises only about 10% of total body fat, it is strongly correlated with increased morbidity and mortality [5], [6].

Recently, V-WAT has been further classified based on drainage, distinguishing “true” V-WAT depots, e.g. omental and mesenteric fat drained by the portal vein, from intra-abdominal (“non-visceral”) depots, drained by the inferior vena cava, including perigonadal (parametrial in females, epididymal in males), retroperitoneal, and perirenal fat [7], [8]. The *portal theory* suggests that elevated hepatic delivery of free fatty acids and proinflammatory cytokines drained from V-WAT via the portal vein, is responsible for insulin resistance and metabolic deterioration in obese individuals [9]. This has been important in demarcating depots that may be more associated with the development of metabolic syndrome. However, even with this delineation, studies using V-WAT are inconsistent in their choice of V-WAT depot. Different fat pads are often used interchangeably although distinct depots are reportedly unique in tissue dynamics (hypertrophic versus hyperplastic response to excess calories), adipokine release, hormonal responses, vascularization, innervation, and abundance of non-adipocyte components [7], [8]. Due to this diversity, it is possible that individual fat pads play differential roles in the pathogenesis of specific diseases. Thus, it is critical that the inherent differences between intra-abdominal fat depots are properly characterized instead of designating one to represent the contribution of all V-WAT.

Although there is no human counterpart, perigonadal fat is often utilized in murine studies of V-WAT-associated diseases, primarily due to its ease of access and relative abundance [8]. However, the use of this depot as representative of all V-WAT may not provide a complete picture and makes drawing depot-specific conclusions difficult. A more comprehensive characterization of each depot is warranted. In support of this, research has demonstrated that differences in fat pad composition and functionality endure after transplantation to different anatomical sites, indicating that other factors, such as SVF content, may contribute to functional heterogeneity [10]. While many studies focus on WAT transcriptomes, proteomes or secretomes, limited information exists on the cellular composition of individual depots. Given the metabolic and immunological relevance of V-WAT, there is clearly a need for elucidation of *depot-specific* SVF composition and gene expression profiles in order to provide a more comprehensive understanding of these distinct microenvironments within the peritoneal cavity. Here, we utilized fluorescence-activated cell sorting (FACS) to comparatively characterize the SVF of parametrial WAT (pmWAT), retroperitoneal WAT (rpWAT), omental WAT (omWAT), and the peritoneal serous fluid (PSF), to determine whether they represent unique microenvironments. We also performed qRT-PCR to assess differences in the gene expression profiles. Our results suggest that pmWAT, rpWAT, omWAT, and PSF represent distinct microenvironments with unique cellular composition in the homeostatic state, supporting our hypothesis that distinct fat depots possess inherent properties that may differentially impact disease states.

## Methods and Procedures

### Ethics Statement

Mice were used in accordance with the guidelines of the Virginia Tech (VT) Institutional Animal Care and Usage Committee (IACUC).

### Animals

Female C57/BL6 mice (Harlan Laboratories) were housed five per cage in a controlled environment (12 hour light/dark cycle at 21°C) with free access to water and food (18% protein rodent chow, Teklad Diets). Mice were sacrificed at 24 weeks of age (27 g average body weight) by CO_2_ asphyxiation.

### Adipose Tissue and Peritoneal Serous Fluid Harvest

OmWAT, pmWAT and rpWAT were harvested from each mouse, weighed, and rinsed with calcium- and magnesium-deficient phosphate buffered saline (PBS^−/−^). OmWAT is attached posteriorly to the stomach, connected to the pancreas and the anterior of the spleen [11]. To ensure that there was no pancreatic contamination, omWAT samples were also tested for buoyancy [12]. PmWAT, the largest fat depot, is comprised of two fat pads, which are located directly under the muscle wall on the dorsal side of the abdomen and attached to the uterine horns. RpWAT also contains two fat pads, which are attached dorsally to the peritoneum, directly behind each kidney. Each tissue was then processed for FACS or placed into RNAlater (Qiagen) and stored at −80°C. Resident peritoneal cavity cells were collected via peritoneal lavage with 5 ml of PBS^−/−^. The effluent was centrifuged, subjected to erythrocyte lysis (155 mM NH_4_Cl, 10 mM KHCO_3_, 0.1 mM EDTA) [2], and further processed as described below.

### Tissue Digestion

SVF from individual fat depots (n = 10) were isolated from digested tissue [13], [14] with minor modifications to improve yields. OmWAT was digested in GKN-buffer containing 1.8 mg/ml type IV collagenase, 10% FBS, and 0.1 mg/ml DNase. The pmWAT and rpWAT digest buffer included a 1∶1 ratio of Krebs-Ringer bicarbonate buffer and collagenase solution (1 mg type I collagenase, 10 mg BSA, and 2 mM CaCl_2_ in 1 ml PBS). Following digest at 37°C for 45 min, cells were passed through a 40 µm cell strainer, and erythrocytes were lysed.

### FACS Analysis

Cell suspensions were washed in flow buffer (2% BSA in PBS^−/−^), blocked with Fc block (BD Biosciences) for 10 minutes at 4°C, rinsed and subsequently incubated with fluorochrome-labeled antibody combinations (available upon request) for 20 min at 4°C. Fluorochrome-labeled antibodies specific for mouse CD45, CD11b, CD11c, F4/80, Ly6C, MHCII, CD34, CD31, CD4, CD44, CD62L, CD25, CD69, B220, CD19, NK1.1, CD73, Flk-1 and Ly6G were obtained from eBioscience (San Diego, CA). CD105 and Ly6a/e antibodies were obtained from BioLegend (San Diego, CA) and Ly6G, CD3, CD8, CD80, CD117 and MR antibodies were obtained from BD Biosciences (San Jose, CA). Prior to analysis, cells were washed twice and resuspended in PBS^−/−^ with propidium iodide for dead cell exclusion. FACS was performed on a FACSAria (BD Biosciences) and data was analyzed using Flowjo (TreeStar) software.

### RNA Extraction

WAT was homogenized in Qiazol (Qiagen), and RNA was purified using an RNeasy Lipid Tissue Kit (Qiagen), according to manufacturer’s instructions. RNA concentration was determined using a NanoDrop1000 spectrophotometer.

### Quantitative Real-time PCR (qRT-PCR)

RNA (n = 6 per tissue) was subjected to the iScript cDNA synthesis system (Biorad) according to manufacturer’s protocol. qRT-PCR was performed with 12.5 ng cDNA per sample using gene-specific SYBR Green primers (primer sequences are available upon request) designed with Beacon Design software. SensiMix SYBR and Fluorescein mastermix (Bioline) was used in a 15 µL reaction volume. qRT-PCR was performed for 42 cycles at 95°C for 15 sec, 58–60°C for 15 sec, and 72°C for 15 sec, preceded by a 10 min incubation at 95°C on the ABI 7900HT (Applied Biosystems). Melt curves were performed to ensure fidelity of the PCR product. L19 was utilized as the housekeeping gene and the ddCt method [15] was used to determine fold differences.

### Statistical Analysis

Data was expressed as mean ± standard error of mean (SEM). FACS and qRT-PCR data were analyzed using a one-way ANOVA coupled with a Tukey post-hoc test in Graphpad Prism. Differences were considered statistically significant at p<0.05.

## Results

To date, studies investigating the contribution of V-WAT to various diseases have often used intra-abdominal fat pads interchangeably. Considering the reported differences between S-WAT and V-WAT, we believe it is important to determine whether intra-abdominal fat pads are indeed similar with respect to their SVF cellular composition, or represent unique signaling microenvironments that may differentially impact intra-peritoneal processes. Here, we use pmWAT due to its widespread application in murine studies, omWAT due to its classification as a “true” visceral fat and importance in immunological surveillance, and rpWAT due to its characterization as a non-visceral intra-abdominal fat depot with a human counterpart.

Seven-month old female C57/BL6 mice were chosen to match the endpoint of many WAT studies that place young mice on specialized treatment regimens for several weeks or months[8], [16]–[19]. This age also allows sufficient fat accumulation for comprehensive FACS analysis of the SVF. Additionally, female mice were utilized because of gender-related differences in adipose accumulation and the importance of omWAT in gynecological diseases, such as ovarian cancer [11], [17].

### Fat Depot Size and Cellularity

**Figure 1:**
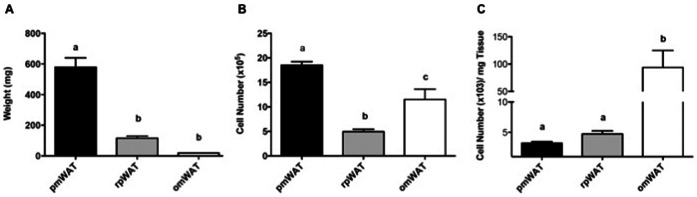
Adipose tissue depot weight and SVF cell counts. (**A**) Whole tissue weight. (**B**) Total number of SVF cells isolated from the digestion of each tissue. (**C**) Number of SVF cells isolated from the digestion of each milligram (mg) of adipose tissue. pmWAT; parametrial WAT, rpWAT; retroperitoneal WAT, omWAT; omental WAT. ^a,b,c^ Unlike letters indicate significance, *p*<0.05.

As expected, pmWAT was the largest fat depot while omWAT represented the smallest (Figure 1A). Despite its significantly smaller size, omWAT yielded total numbers of SVF cells similar to pmWAT, whereas rpWAT had a significantly smaller SVF population (p<0.0001) (Figure 1B). Thus, 20 times more SVF cells *per milligram of tissue* were isolated from omWAT, as compared to pmWAT (p = 0.009) or rpWAT (p = 0.01) (Figure 1C).

### Fat Depot SVF Characterization

**Figure 2:**
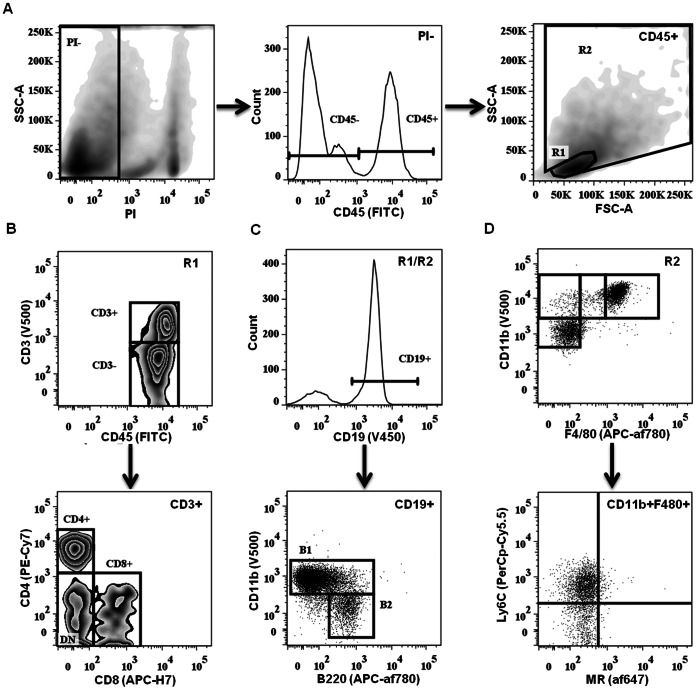
Flow cytometric analysis gating strategy. (**A**) Digested tissue samples were subjected to PI live/dead cell exclusion and CD45^+^ leukocytes were divided into R1 (lymphocytes) and R2 (mono-, granulocytes) gates based on forward/side scatter followed by doublet exclusion. (**B**) CD3^+^ T cells from R1 were further subclassified either as CD4^+^ T_H_, CD8^+^ T_C_ or DN (double negative). (**C**) CD19^+^ B cells from R1 and R2 were further subclassified into CD11b^+^ B1 or CD11b^−^ B2. (**D**) Monocytic populations from R2 were classified based on CD11b, F4/80 and CD11c (not shown) staining, and further subdivided based on activation markers.

Individual fat depots were characterized via FACS analysis to identify depot-specific differences. PSF was included as an established immunologically active microenvironment present within the peritoneal cavity [20], [21]. Leukocyte subsets were identified based on well-defined surface markers (Figure 2). First, viable cells (identified via propidium iodide exclusion) were separated into CD45^+^ leukocytes and CD45^−^ stromal constituents. The CD45^+^ population was subsequently separated into R1 (lymphocyte) and R2 (monocyte/granulocyte) gates based on forward/side scatter (Figure 2A). T-lymphocyte subsets within the CD3^+^ fraction (R1) were further separated into CD4^+^ T-helper (T_h_) cells, CD8^+^ T-cytotoxic (T_c_) cells, NK1.1^+^ natural killer T-cells (NKT), or CD4^−^CD8^−^ double-negative (DN) cells (Figure 2B). CD19^+^ B-cells, distributed within both the R1 and R2 gates, were gated into B1 (B220^lo/+^CD11b^+^) and B2 (B220^lo/+^CD11b^−^) subsets (Figure 2C). Monocyte/granulocyte populations (R2) were classified based on CD11b, CD11c and F4/80 staining, followed by analysis of additional surface markers (Ly6C, Ly6G, mannose receptor (MR), CD80, CD69 and CD93) (Figure 2D).

**Figure 3:**
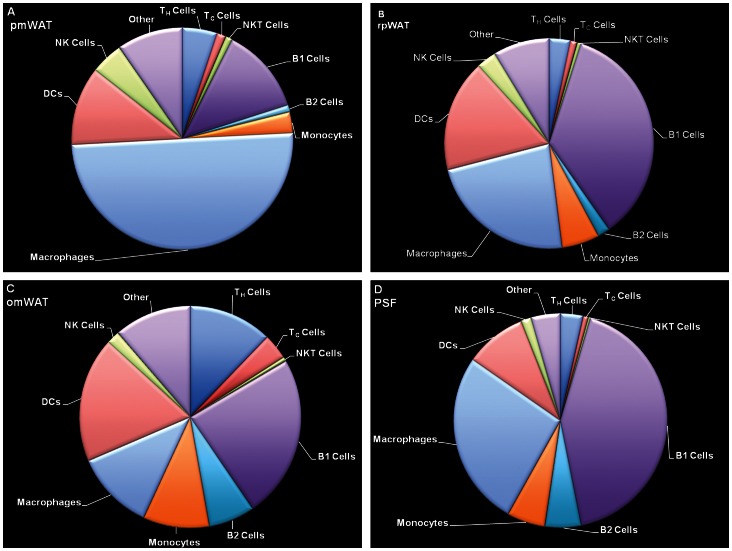
Flow cytometric analysis of leukocyte populations in the SVF of (A) pmWAT, (B) rpWAT, (C) omWAT and (D) PSF. Natural killer (NK) cells include mNKs and preNKs, dendritic cells (DC) include DC (I) from R1 and DC (II) from R2, macrophages include large (LPMs) and small (SPM) peritoneal macrophages, monocytes include monocytes (I) (CD11b^+^F4/80^−^) and monocytes (II) (CD11b^lo^F4/80^−^) populations, and “other” includes regulatory T cells (T_reg)_, CD3^+^CD4^−^CD8^−^NK1.1^−^ subset, and myeloid precursors from R1, as well as neutrophils (PMNs), and pre-B or –macrophage cells (PreBoMs) from R2.

Markers defining specific populations as well as distribution of leukocyte subsets within the respective fat pads are detailed in Figure 3 and Table 1. The compositional profiles of individual fat depots were clearly different, supporting the hypothesis that each fat depot is a unique microenvironment harboring distinct immune cell populations. All three fat depots contained a large CD45^−^ population within the SVF, whereas the PSF was limited to CD45^+^ leukocytes (99.1%). Consistent with its role as a secondary lymphoid organ, omWAT contained a two-fold higher CD45^+^ population than pmWAT and rpWAT (p<0.001).

**Table 1:** Leukocyte Characterization of Immune Microenvironments.

Cell Type (origin)	Markers	pmWAT	rpWAT	omWAT	PSF
CD45^+^ (of viable)		38.1 (3.6)	38.1 (2.5)	75.4 (1.2)	99.1 (0.3)
Lymphocytes (R1)		29.0 (3.1)	29.1 (3.0)	53.0 (8.3)	62.0 (1.1)
Mono/Granulocytes (R2)		62.2 (3.0)	61.1 (3.5)	39.3 (8.3)	34.7 (3.1)
B1-B	CD19^+^CD11b^+^B220^lo/+^	11.5 (1.7)	40.0 (2.1)	25.0 (1.1)	38.1 (1.2)
B2-B	CD19^+^CD11b^-^B220^lo/+^CD5^−^	0.9 (0.2)	2.2 (0.4)	7.1 (0.9)	4.9 (0.8)
T_H_	CD3^+^CD4^+^	4.6 (0.8)	3.7 (0.6)	12.8 (1.8)	3.1 (0.2)
T_C_	CD3^+^CD8^+^	1.2 (0.1)	1.2 (0.2)	3.9 (0.5)	0.7 (0.1)
NKT	CD3^+^NK1.1^+^	0.9 (0.1)	0.7 (0.1)	0.6 (0.1)	0.3 (0.1)
T_REG_	CD3^+^CD4^+^CD25^+^	0.8 (0.3)	1.5 (0.4)	2.5 (0.2)	1.6 (0.2)
CD3^+^CD4^−^CD8^−^NK1.1^−^		2.4 (0.2)	2.3 (0.4)	4.5 (0.4)	0.4 (0.1)
Myeloid precursors	CD3^−^B220^−^NK1.1^−^CD11b^hi^	5.1 (0.6)	5.2 (0.4)	4.8 (0.3)	2.0 (0.1)
mNK	NK1.1^+^CD11b^+^CD3^−^	3.4 (0.3)	3.0 (0.3)	1.4 (0.2)	1.3 (0.1)
preNK	NK1.1^+^CD11b^-^CD3^−^	0.9 (0.1)	0.5 (0.1)	0.6 (0.1)	0.1 (0.04)
DCs(I)	CD11c^+^CD11b^lo/+^	6.7 (0.4)	8.3 (0.5)	7.6 (0.7)	4.6 (0.4)
DCs (II)	CD11b^+/lo^CD11c^+^F4/80^−^	3.7 (1.5)	11.3 (6.4)	11.5 (4.0)	3.8 (1.5)
LPMs	CD11b^+^F4/80^+^	3.6 (0.6)	3.1 (0.5)	1.1 (0.2)	21.0 (1.4)
SPMs	CD11b^+^F4/80^lo^	41.6 (2.1)	22.9 (1.2)	11.1 (1.2)	2.9 (1.1)
Monocytes (I)	CD11b^+^F4/80^−^	1.4 (0.6)	2.2 (3.8)	4.0 (1.7)	0.2 (0.1)
Monocytes (II)	CD11b^lo^F4/80^−^	1.4 (1.1)	4.4 (1.4)	6.1 (2.4)	5.0 (2.2)
PMNs	CD11b^+^Ly6G^+^Ly6C^+^	0.4 (0.1)	0.8 (0.1)	0.04 (0.03)	0
PreBoMs	CD19^+^CD11b^hi^B220^lo^F480^+^CD93^+^CD69^+^	0	0	0	0.7 (0.1)

Data are presented as % of total CD45^+^ (+/− standard error of the mean). pmWAT, parametrial fat; rpWAT, retroperitoneal fat; omWAT, omental fat; and PSF, peritoneal serous fluid. T_h_, Helper T cells; T_c_, cytotoxic T cells; NKT, natural killer T cells; T_reg,_ regulatory T cells; DCs, dendritic cells; LPMs, large peritoneal macrophages; SPMs, small peritoneal macrophages; PMNs, neutrophils; and PreBoMs, pre-B or –macrophage cells.

#### Overall distribution

Of total compartmental leukocytes, pmWAT contained the highest proportion of macrophages (45.2%), with smaller populations of B-cells (12.9%), T-cells (8.2%) and monocyte subsets (2.8%) (Figure 3, Table 1). The immune composition of rpWAT displayed distinct similarities (larger B1- and dendritic cell (DC) and monocyte populations) to omWAT whereas T- and natural killer (NK) cell frequencies (7.3%; 3.5%) more closely matched those of pmWAT, its non-visceral counterpart. OmWAT had proportionally more T-cells (21.5%), DCs (19.1%) and B-cells (35.5%) than pmWAT or rpWAT (Figure 3). Confirming previous reports [20], PSF contained a large proportion of B1-cells (38.1%), macrophages (21.0%) and DCs (8.7%), indicative of ongoing immunosurveillance in the peritoneal cavity (Figure 3, Table 1).

#### Lymphocyte characterization

OmWAT contained the highest proportion of T-cells, consistent with its role in antigen presentation and development of cell-mediated responses [22]. This corresponds with high expression of *Ccl21*, a chemoattractant important in the homing of T-cells to lymphoid organs. PmWAT and rpWAT had three-fold fewer T-cells (p<0.0001) than omWAT, although the T_h_:T_c_ ratio in all four microenvironments was 4∶1. There were no significant depot-specific differences within the memory (CD44+/CD62L^+/−^) or naïve (CD44^−/^CD62L^+^) T_h_ or T_c_ subsets (data not shown). The proportion of NKT-cells was significantly lower in PSF than all fat depots (p<0.001). Interestingly, the majority (>60%) of NK-cells within all four compartments were CD94^hi^, which has recently been associated with increased proliferation, production of IFNy, and target cell lysis [23]. Further, the CD27^hi^ subset of mature NK (mNK) cells in omWAT was two-fold higher (p<0.001, data not shown) than the other microenvironments. CD27 expression has been linked to increased responsiveness to chemokines and interactions with DCs, again consistent with the role of omWAT as a major peritoneal immunosurveillance organ [24].

#### Monocyte/Granulocyte characterization

**Figure 4:**
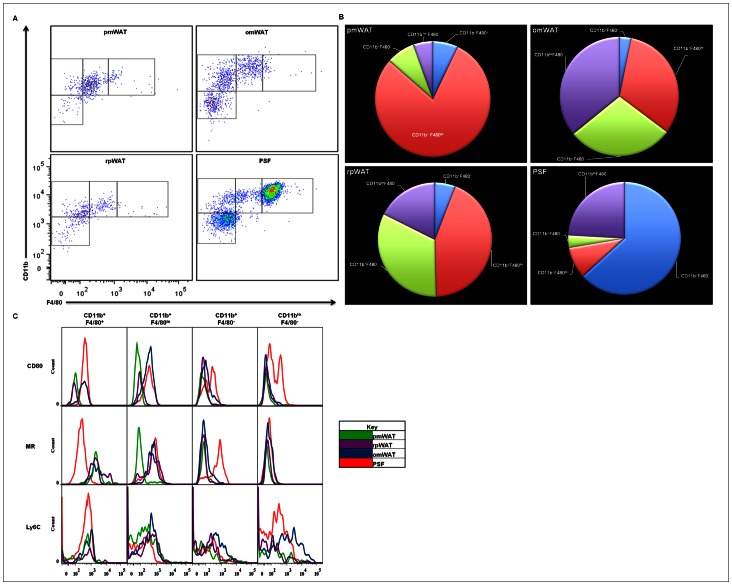
Myeloid populations found in pmWAT, rpWAT, omWAT and PSF. (**A**) Resident myeloid populations differ in a microenvironment-specific manner. (**B**) Differential CD11b and F4/80 expression displayed as a percentage of total R2 gate. (**C**) Activation status of respective myeloid populations differ in a microenvironment-specific manner.

Within the R2 gate, four populations based on relative CD11b and F4/80 expression were discernible. Previous reports have described two functionally distinct macrophage subsets within the PSF: the “large peritoneal macrophages” (LPMs), named for their increased forward/side scatter (predominant in the homeostatic state), and “small peritoneal macrophages” (SPMs), which increase in number following LPS stimulation [25]. The largest R2 population present within the PSF was the CD11b^+^F4/80^+^-LPMs, (61.7%), followed by CD11b^+^F4/80^lo^-SPMs, CD11b^+^F4/80^–^monocyte (I) and CD11b^lo^F4/80^lo^-monocyte (II) populations present at 8.3%, 3.5%, and 23.5%; respectively (Figure 4A, 4B).

In contrast, the predominant R2 population in pmWAT was SPMs (77.7%), with minor populations of LPMs (7.0%) and monocytes (I and II) (7.6%; 5.4%). Similarly, in rpWAT the SPM population was predominant (42.4%), with LPMs representing only 5.7% of total cells within the R2 gate. However, the monocytic (I and II) subsets represented a significant proportion (31.7%, and 17.1%; respectively) of the R2 gate. The omWAT R2 composition was also defined by a predominant SPM subset (30.9%), with the LPMs and monocytes (I and II) comprising 3.1%, 27.9%, and 34.6%; respectively.

A CD80/MR expression profile was used to evaluate depot-specific differences in macrophage activation status. SPMs residing within PSF and omWAT expressed CD80, whereas pmWAT and rpWAT SPMs were CD80^−^. MR expression was only noted in SPMs isolated from PSF, omWAT and rpWAT (Figure 4C). This further suggests that depot-specific factors inherently contribute to activation or differentiation of their inherent SVF leukocytes.

#### PreBoMs

**Figure 5:**
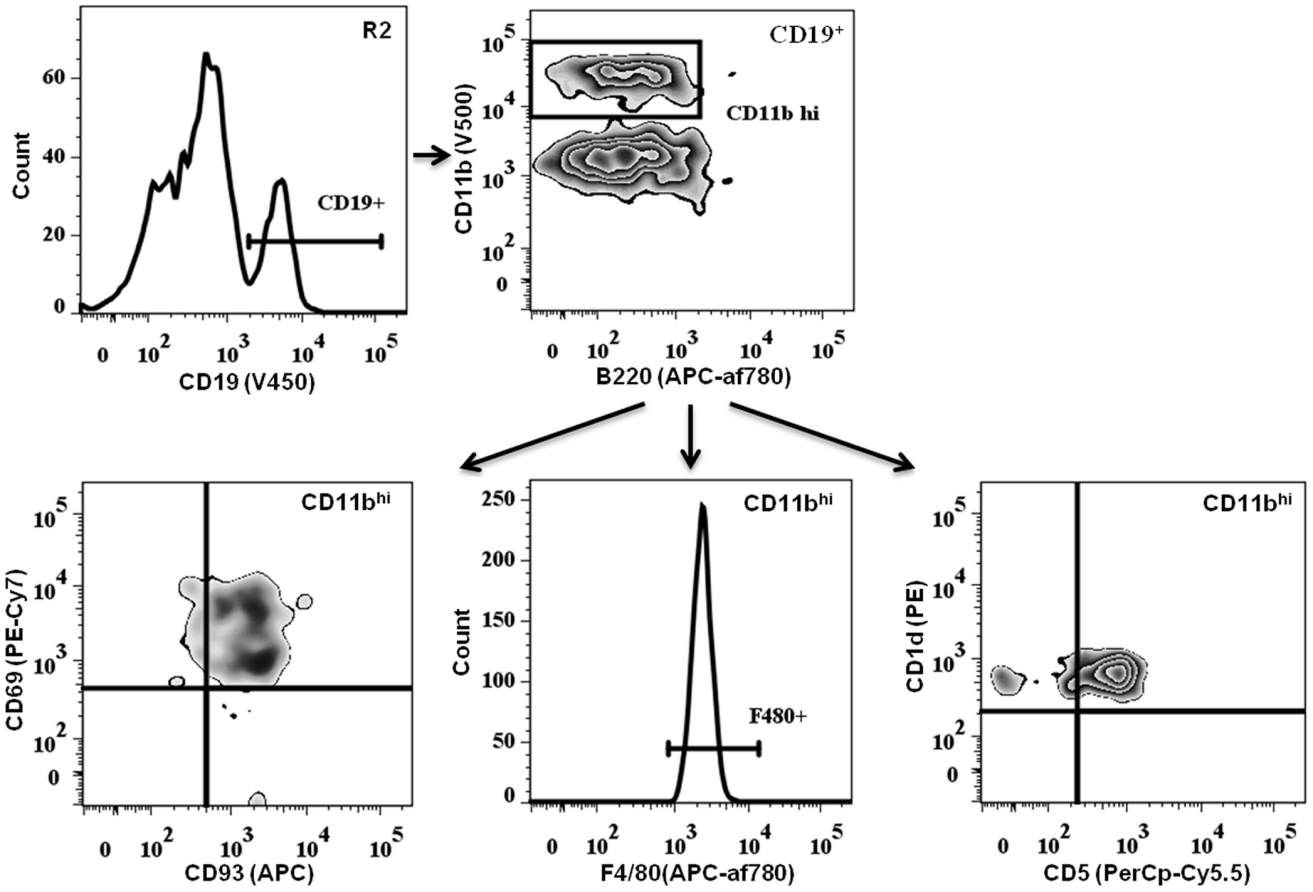
Identification of a unique CD45+ population in the PSF. Pre-B or –macrophage cells (PreBoMs) found within the R2 gate express CD19^+^CD11b^hi^F480^+^CD93^+^CD69^+^CD1d^+^CD5^+^ phenotype. This subset was not found in WAT.

During analysis, a small, distinct CD45^+^ cell population (0.7%) was identified within the PSF that was not present in the fat depots. We refer to this subset as “PreBoMs” (pre-B or -macrophages) due to their expression of unique and potentially novel surface markers (Figure 5). These cells were CD19^+^B220^lo/+^, expressed high levels of CD11b and were found within the R2 gate, implying a more granular phenotype. Additionally, they expressed CD93, a premature B-cell marker, and CD69, an early activation marker. Similar to the age-associated and IL-10-producing B-cells reported recently [26], [27], preBoMs were CD1d^+^ and CD5^+^. However, they also expressed F4/80, a mature macrophage marker. We were unable to evaluate CD11c expression within this population to confirm if these cells are one of the aforementioned novel B-cell subtypes, or a new subset residing within the peritoneal cavity. Because of the importance of innate-like B-cell subsets in peritoneal immunosurveillance, it needs to be determined whether this subset plays a role in metabolic disorders and peritoneal diseases.

#### Progenitor/Stem populations

**Figure 6:**
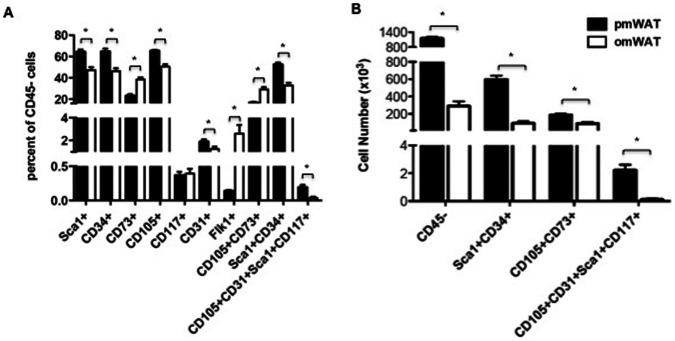
Flow cytometric analysis of stem and progenitor cell markers. (**A**) Percentage of CD45^−^ cells that are positive for indicated markers. (**B**) Total number of SVF cells positive for selected markers in pmWAT and omWAT.

We also examined the prevalence of stem and progenitor cells as they may contribute to microenvironmental signaling and tissue-specific responses to energy. Additionally, they may be recruited and educated to participate in tissue repair and re-organization and may contribute to various disease states. Figure 6A provides an overview of some notable progenitor markers present on CD45^−^ cells within pmWAT and omWAT. RpWAT did not contain a sufficient SVF content for analysis of both immune and progenitor populations in this tissue.

CD34, CD73, CD105, Sca1, CD31, and Flk1 were all differentially expressed in pmWAT and omWAT (p<0.05) (Figure 6A). Of note, CD45^−^CD105^+^CD73^+^ mesenchymal stem cells (MSCs) [28] were a more prominent percentage of the SVF in omWAT (p<0.0001), while CD45^−^Sca1^+^CD34^+^ adipocyte precursor cells (APCs) [29] and CD45^−^CD105^+^CD31^+^Sca1^+^CD117^+^ endothelial progenitor cells (EPCs) [30] were a higher percentage in pmWAT (both p<0.0001). However, based on total cell numbers, pmWAT contained a greater number of all three subsets due to the higher proportion of CD45^−^ cells within the SVF (61.9% versus 24.5% in pmWAT and omWAT, respectively, p<0.001) (Figure 6B). Thus, stem and progenitor populations may also contribute to depot-specific differences between intra-abdominal adipose tissues.

### Fat Depot Specific mRNA Expression Profile

qRT-PCR was performed to more extensively characterize the overall adipose tissue signaling milieu in these unique microenvironments. It is important to note that expression patterns represent the tissue as a whole (except in PSF), and thus are reflective of both adipocytes and SVF. A panel of inflammatory mediators, angiogenesis-associated molecules, adipokines and lipolysis-associated enzymes were used to provide an overview of genes that may contribute to depot-specific differences.

**Figure 7:**
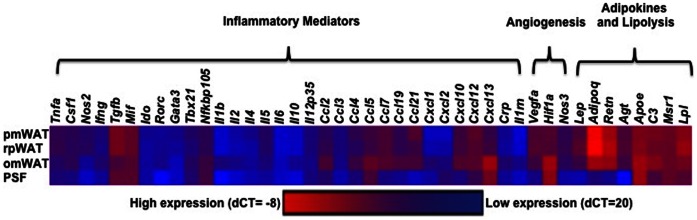
Heat map of mRNA expression profiles of adipose tissue depots (based on dCT values).

RpWAT and pmWAT exhibited highly similar mRNA expression profiles (Figure 7, Table 2), potentially due to the prevalence of adipocytes and CD45^−^ cells. In contrast, significantly higher (p<0.05) expression of cytokines (*Il-1b*, *Il-2*, *Il-10*) and chemokines (*Ccl2*, Ccl5, *Ccl7*, *Ccl19*, *Cxcl1*, *Cxcl2*, *Cxcl10*, *Cxcl13*) was observed in omWAT. This may be due in part to the large resident leukocyte population and it may also be required for active recruitment and continual maintenance of high leukocyte numbers. OmWAT also expressed significantly higher levels of *Apoe*, and lower levels of *Adipoq*, *Agt,* and *Lpl* as compared to pmWAT and rpWAT, indicating differences in lipid homeostasis regulation. Additionally, omWAT displayed increased *Hif1a* expression, which may be the result of chronic hypoxia [11]. However, this was not associated with a concomitant increase in *Vegfa*, perhaps because *Vegfa* was already highly expressed.

**Table 2:** Gene Expression Profiles of Immune Microenvironments.

Gene	rpWAT	omWAT	PSF
*Tnfa*	0.50 (0.67)	−3.84 (0.39)*	−9.17 (1.70)*
*Csf1*	−1.04 (0.52)	−0.90 (0.47)	−11.84 (2.05)*
*Nos2*	1.14 (0.65)	−17.74 (4.02)*	−134.99 (30.80)*
*Ifng*	−1.55 (1.24)	0.26 (0.68)	−7.76 (1.712)*
*Tgfb*	−1.27 (.14)	−23.33 (1.26)*	−7.84 (0.99)*
*Mif*	0.01 (0.62)	−2.25 (0.22)*	−1.07 (0.77)
*Ido*	−0.71 (1.01)	−1.61 (0.25)	−1.02 (0.76)
*Rorc*	1.38 (1.94)	1.66 (0.90)	−17.99 (0.50)*
*Gata3*	−1.69 (0.81)	−8.80 (2.92)*	−13.99 (2.46)*
*Tbx21*	−1.10 (0.93)	1.39 (0.65)	−0.84 (1.02)
*NFkB*	−0.31 (0.74)	3.02 (0.24)*	4.62 (1.02)*
*Il1b*	0.20 (1.12)	114.42 (78.18)*	9.57 (7.71)
*Il2*	−1.80 (1.57)	6.36 (1.49)*	−6.19 (2.18)*
*Il4*	−6.31 (4.47)	−1.57 (0.72)	−6.10 (1.81)*
*Il5*	−0.84 (0.85)	−3.38 (0.59)*	−24.54 (7.77)*
*Il6*	−0.55 (0.69)	1.86 (0.70)	0.85 (0.85)
*Il10*	−1.00 (0.54)	9.62 (1.73)*	−2.58 (0.62)*
*Il12a*	0.50 (0.66)	−1.88 (0.25)	−0.31 (0.88)
*Ccl2*	2.29 (1.53)	67.59 (33.34)*	−2.80 (1.64)
*Ccl3*	−0.85 (0.81)	−1.82 (0.67)	14.30 (5.41)*
*Ccl4*	−2.44 (0.50)	2.39 (0.35)	−14.10 (4.18)*
*Ccl5*	−1.00 (0.50)	11.65 (2.08)*	2.73 (1.08)*
*Ccl7*	0.52 (0.97)	1.86 (2.0)*	−214.80 (112.99)*
*Ccl19*	−0.47 (0.80)	5.76 (0.90)*	−42.96 (10.35)*
*Ccl21*	−10.27 (5.81)*	−3.46 (1.60)	−4387.09 (1360.31)*
*Cxcl1*	2.95 (0.56)	54.61 (20.49)*	5.52 (2.07)*
*Cxcl2*	3.36 (0.90)	32.22 (15.48)*	96.50 (37.33)*
*Cxcl10*	−0.089 (0.87)	4.72 (0.86)*	−46.87 (12.43)*
*Cxcl12*	−2.32 (0.42)*	−0.98 (0.51)	−21.79 (3.19)*
*Cxcl13*	2.06 (1.08)	488.67 (56.84)*	67.45 (19.31)*
*Crp*	−2.18 (1.69)	22.73 (13.83)*	4.03 (1.77)
*Prom1*	0.51 (0.77)	−0.15 (1.10)	−17.06 (4.83)
*IL1rn*	1.27 (2.10)	50.39 (24.96)*	3.31 (0.99)
*Vegfa*	1.05 (0.53)	−1.64 (1.62)	−255.32 (31.63)*
*Hif1a*	−0.89 (0.51)	74.87 (10.38)*	33.99 (5.56)*
*Nos3*	1.25 (0.60)	−2.25 (0.52)	−112.30 (40.96)*
*Lep*	3.65 (1.19)	−4.62 (2.16)	−1233.93 (376.92)*
*Adipoq*	1.78 (0.24)	−31.56 (5.28)*	−287768.13 (57680.49)*
*Retn*	2.25 (0.20)*	−2.08 (0.45)	−28554.69 (8773.49)*
*Agt*	1.29 (0.13)	−3.14 (0.77)*	−31365.74 (0.00)*
*Apoe*	1.50 (0.26)	5.64 (0.46)*	5.55 (1.63)*
*C3*	−1.75 (0.85)	2.49 (0.38)	−17.65 (5.20)*
*Msr1*	−1.32 (0.14)	−0.99 (0.46)	−1.43 (0.67)
*Lpl*	2.21 (0.25)*	−3.76 (1.10)	−22.46 (0.74)

Data are presented as fold changes compared to pmWAT (± standard error of the mean), *p<0.05.

PSF, which was comprised almost exclusively of CD45^+^-leukocytes, exhibited an mRNA expression profile similar to omWAT, denoted by decreased expression of *Tnfa* and *Il5*, and increased expression of *Ccl5, Cxcl1, Cxcl2, Cxcl13,* and *Tgfb* as compared to pmWAT. However, PSF expression of *Csf1, Ifng, Rorc, Il4, Ccl4, Ccl7, Ccl21, Cxcl12, Vegfa, C3*, and *Nos3* were significantly lower than all fat depots. Additionally, *Il2, Il10, Ccl19*, and *Cxcl10* were significantly higher (p<0.05) in omWAT, but were significantly lower (p<0.05) in PSF as compared to pmWAT. As anticipated, of the three fat depots PSF was most similar to omWAT, which acts as its filtration system. However, as PSF is composed of primarily leukocytes, the cellular components and interactions among cell types in the two microenvironments are very different, resulting in unique expression profiles.

## Discussion

While V-WAT has been associated with various metabolic disorders and certain cancers [1], [5], [6], the exact contribution of individual V-WAT depots to physiological and pathological functions is poorly understood. Here, we characterized SVF composition and homeostatic gene expression profiles of three intra-abdominal fat pads to determine depot-specific differences, as this may critically impact their functionality. This study highlights the unique immune profile of intra-abdominal fat depots and supports our hypothesis that they may each have distinct roles in biological functions and disease pathogenesis and thus should be evaluated independently.

In agreement with the previous designation of pmWAT and rpWAT as non-visceral intra-abdominal fat pads, the initial compositional profile of leukocytes based on lymphocyte (R1) versus monocyte/granulocyte (R2) fractions revealed an identical 1∶2 ratio, while omWAT and PSF displayed a ratio of 3∶2. This indicates that lymphocytes are the predominant leukocyte population in omWAT and PSF, while the monocyte/granulocyte fraction is dominant in pmWAT and rpWAT. However, upon further evaluation, high numbers of B1-cells were found within the R2 gate of rpWAT. Although the R1:R2 ratios of pmWAT and rpWAT are very similar, when expressed as a proportion of total T- and B-cells, the immune cell composition of rpWAT more closely resembles that of omWAT and PSF. This was unexpected as the omWAT is considered “true” V-WAT while rpWAT has been described as a non-visceral intra-peritoneal fat pad.

B1-lymphocytes play an important role in immunosurveillance in the peritoneal cavity and are considered “innate-like” B-cells, possessing pattern recognition receptors to conserved bacterial and viral epitopes [31]. In naïve animals, there is a constant flux of B1-cells between omWAT and the PSF. However, their presence in other intra-abdominal fat pads has not been reported. Unexpectedly, we found a large proportion of B1-cells in rpWAT, comparable to PSF and higher than omWAT. Additionally, rpWAT expressed higher levels of *Il5*, a stimulator of B-cell growth and immunoglobulin secretion [32], than omWAT. In concordance with FACS data, qRT-PCR indicated constitutively high expression of *Cxcl13*, a chemoattractant for B1-cells [33], in all four microenvironments. Together, this suggests that the rpWAT may serve a yet undefined function in peritoneal B-cell immunomodulation.

An unexpected finding during the course of this study was the appearance of CD19^+^F4/80^+^B220^lo^CD11b^hi^CD1d^+^CD5^+^D93^+^CD69^+^ cells, which we have named PreBoMs based on their expression of B-cell-, macrophage- and progenitor-specific markers. It is unclear if this population represents the IL-10-producing regulatory (recently reported in spleen and PSF) [34], [35] or age-associated B-cells (reported in spleen only) [36], since the expression of F4/80 has not been reported in either cell type. Further analyses are needed to determine whether this PreBoM population is indeed a novel B-cell-like subset within the peritoneal cavity.

The CD45^−^ component of the SVF includes fibroblasts, endothelial cells, and stem and progenitor populations. Adipose-associated stem and progenitor cells have been implicated in various biological processes and pathological conditions [37], [38]. While not comprehensive, our analysis highlights significant depot-specific stem/progenitor populations present in pmWAT and omWAT. The higher numbers of MSCs, APCs, and EPCs in pmWAT suggest that it may be a more abundant source of recruitable stem/progenitor cells. Similar to fat depot-specific leukocyte trafficking, unique signaling milieus may account for differences in progenitor populations. More in-depth depot-specific analyses are warranted to determine the local and systemic implications of these populations in individual fat depots, particularly in the context of human disease states.

In summary, our results support the hypothesis that intra-abdominal fat pads represent distinct signaling microenvironments, each serving as its own “mini-organ” [39]. In agreement with previous reports indicating important functional differences between true visceral and non-visceral intra-abdominal fat pads in various metabolic disorders, we have found distinct differences in the SVF even within these subgroups. Our comparison of SVF composition, as well as whole-tissue gene expression of pmWAT, rpWAT, and omWAT, indicate that intra-abdominal fat depots cannot be used interchangeably. Hence, this extensive characterization of these immunomodulatory microenvironments provides a foundation for the design of future studies investigating depot-specific impacts on peritoneal homeostasis and chronic diseases. These findings support the need for a new paradigm for the manner in which intra-abdominal fat should be studied.
